# A new white-spotted *Megaselia* Rondani (Diptera: Phoridae) from western North America

**DOI:** 10.3897/BDJ.7.e34310

**Published:** 2019-04-23

**Authors:** Brian V. Brown, Maria A. Wong, Emily Hartop

**Affiliations:** 1 Natural History Museum of Los Angeles County, Los Angeles, California, United States of America Natural History Museum of Los Angeles County Los Angeles, California United States of America; 2 Naturhistoriska Riksmuseet, Stockholm, Sweden Naturhistoriska Riksmuseet Stockholm Sweden; 3 Stockholms Universitet, Stockholm, Sweden Stockholms Universitet Stockholm Sweden; 4 Station Linné, Färjestaden, Sweden Station Linné Färjestaden Sweden

**Keywords:** Diptera, Phoridae, Megaselia, new species, urban biodiversity

## Abstract

**Background:**

The phorid fly genus *Megaselia* Rondani is a large, poorly-known taxon whose species are found worldwide.

**New information:**

A new species of *Megaselia* Rondani, *M. simunorum*, is described from both urban and rural sites in southern California. With a large area of white colour on the posterior part of the abdominal dorsum, it closely resembles the much more common species *M. sulphurizona*, but *M. simunorum* has distinctly thicker ventral setae on the abdomen and a differently-shaped white spot.

## Introduction

The enormous genus *Megaselia* Rondani has many difficult-to-separate species, but a few seem almost immediately identifiable, such as the common western North American species *Megaselia sulphurizona* Borgmeier. This species, although originally described from just eight specimens from California, Washington and Idaho, USA, is widespread within western USA and is one of the most abundant species collected in urban Los Angeles by the BioSCAN project (Brown and Hartop 2016). The original description (Borgmeier 1966) noted that tergites 5 and 6 of the male were wholly or partly “pale yellow”, a character upon which the name was based (translation of *sulphurizona* is loosely “yellow belt”). Borgmeier was working with air-dried specimens, in which colour can be distorted, however and we found that fresh specimens usually have a white spot. Regardless, until now, the identification of *M. sulphurizona* has been extremely straightforward.

Amongst the many thousands of phorid flies captured by Malaise traps in the BioSCAN project were a few specimens of “another” white-spotted species. Furthermore, we found large differences in the extent of the white colour on the dorsum of *M. sulphurizona* and started to explore the variation within this species. We take this opportunity to describe our first, most distinctive, new white-spotted *Megaselia* that, based on its divergent abdominal structure, is apparently not closely related to *M. sulphurizona* (whose systematics we plan to study later).

## Materials and methods

The description of this species follows the reduced, table-based method we previously established (Hartop and Brown 2014,Hartop et al. 2015, Hartop et al. 2016). Specimens were collected in Townes lightweight Malaise traps (Townes 1972) and preserved in 95% alcohol. Some specimens were dried using HMDS (Brown 1993) and glued to insect pins; others were slide-mounted following Disney (2009), except that the permanent mounting medium Canada Balsam was used. Most specimens are stored in the Natural History Museum of Los Angeles County (LACM), although some were placed in the collections of the California Academy of Sciences (CAS) and the California Department of Food and Agriculture (CSCA).

## Taxon treatments

### Megaselia simunorum

Brown, Wong, and Hartop sp. n.

urn:lsid:zoobank.org:act:D300CD48-1374-40A5-BC23-82EDF0E7467C

#### Materials

- **Type status:** Holotype. **Occurrence:** catalogNumber: LACM ENT 366270; sex: male; **Location:** country: USA; stateProvince: California; county: Los Angeles; locality: Encino; verbatimCoordinates: 34.167°N, 118.513°W; verbatimCoordinateSystem: decimal degrees; decimalLatitude: 34.167; decimalLongitude: -118.513; **Event:** eventID: BioSCAN 18490; samplingProtocol: Malaise trap; **Record Level:** institutionCode: LACM; basisOfRecord: Preserved specimen
- **Type status:** Paratype. **Occurrence:** recordedBy: B.Brown, G.Kung; sex: male; lifeStage: adult; **Location:** country: USA; stateProvince: California; county: Kern; locality: Wind Wolves Preserve; verbatimCoordinates: 34.956°N, 119.187°W; verbatimCoordinateSystem: decimal degrees; decimalLatitude: 34.956; decimalLongitude: -119.187; **Event:** samplingProtocol: Malaise trap; verbatimEventDate: 9-15.v.2018; **Record Level:** institutionID: LACM; institutionCode: LACM; basisOfRecord: PreservedSpecimen
- **Type status:** Paratype. **Occurrence:** recordedBy: B.Brown, G.Hendler; sex: male; **Location:** country: USA; stateProvince: California; county: Los Angeles; verbatimLocality: Topanga Canyon; verbatimElevation: 250 m; minimumElevationInMeters: 250; maximumElevationInMeters: 250; decimalLatitude: 34.08; decimalLongitude: -118.60; **Event:** samplingProtocol: Malaise trap; verbatimEventDate: 7-14.iii.1994; **Record Level:** institutionID: LACM; basisOfRecord: PreservedSpecimen
- **Type status:** Paratype. **Occurrence:** recordedBy: B.V.Brown; individualCount: 3; sex: male; lifeStage: adult; **Location:** country: USA; stateProvince: California; county: San Luis Obispo; locality: Los Osos,Pecho Willows; verbatimLocality: Pecho Willows; verbatimElevation: 5 m; decimalLatitude: 35.317; decimalLongitude: -120.853; **Event:** samplingProtocol: Malaise trap; verbatimEventDate: 2-8.vii.2017; eventRemarks: old field near bay; **Record Level:** institutionID: LACM; basisOfRecord: PreservedSpecimen
- **Type status:** Paratype. **Occurrence:** recordedBy: B.V.Brown; individualCount: 3; sex: male; lifeStage: adult; **Location:** country: USA; stateProvince: California; county: San Luis Obispo; locality: Rancho El Chorro; verbatimElevation: 110 m; decimalLatitude: 35.34; decimalLongitude: -120.73; **Event:** samplingProtocol: Malaise trap; verbatimEventDate: 5-8.vii.2017; habitat: forest near stream; **Record Level:** collectionID: LACM; basisOfRecord: Preserved specimen
- **Type status:** Paratype. **Occurrence:** recordedBy: P.H.Arnaud; individualCount: 7; sex: male; lifeStage: adult; **Location:** country: USA; stateProvince: California; county: San Mateo; locality: Palo Alto, Stanford University; **Event:** samplingProtocol: Malaise trap; verbatimEventDate: 1-15.i.1995; **Record Level:** institutionID: CAS, CSCA, LACM; basisOfRecord: PreservedSpecimen
- **Type status:** Paratype. **Occurrence:** recordedBy: P.H.Arnaud; individualCount: 3; sex: male; lifeStage: adult; **Location:** country: USA; stateProvince: California; county: San Mateo; locality: Palo Alto, Stanford University; **Event:** samplingProtocol: Malaise trap; verbatimEventDate: 26-31.xii.1994; **Record Level:** institutionID: LACM; basisOfRecord: PreservedSpecimen

#### Description

See Table 1. A CO1 barcode is deposited in the BOLD database as BOLD: ADK7956.

#### Diagnosis

**Figure 4.**
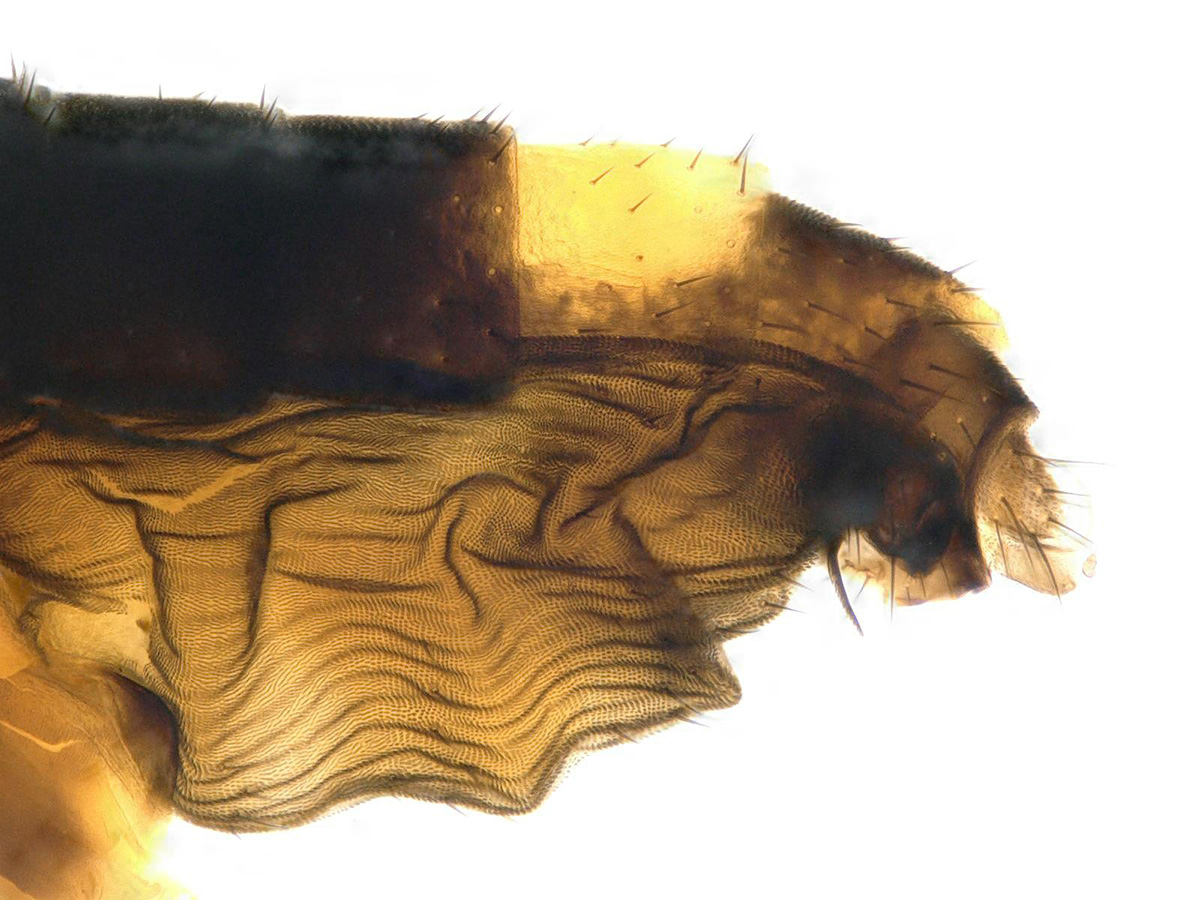
*Megaselia sulphurizona* Borgmeier, male abdomen, lateral.

This species differs from all North American *Megaselia*, except those similar to *M. sulphurizona*, by the contrasting white colour of the posterior abdominal tergites. The lighter coloured halter, stronger ventral abdominal setae (compare with *M. sulphuriza*, Fig. 4) and the reduced tergite 5 with large posterior setae further distinguish this species.

#### Etymology

Named in memory of Dr. Patricia Bates Simun and Mr. Richard V. Simun by their daughters, Ann and Mary.

#### Distribution

**Figure 6.**
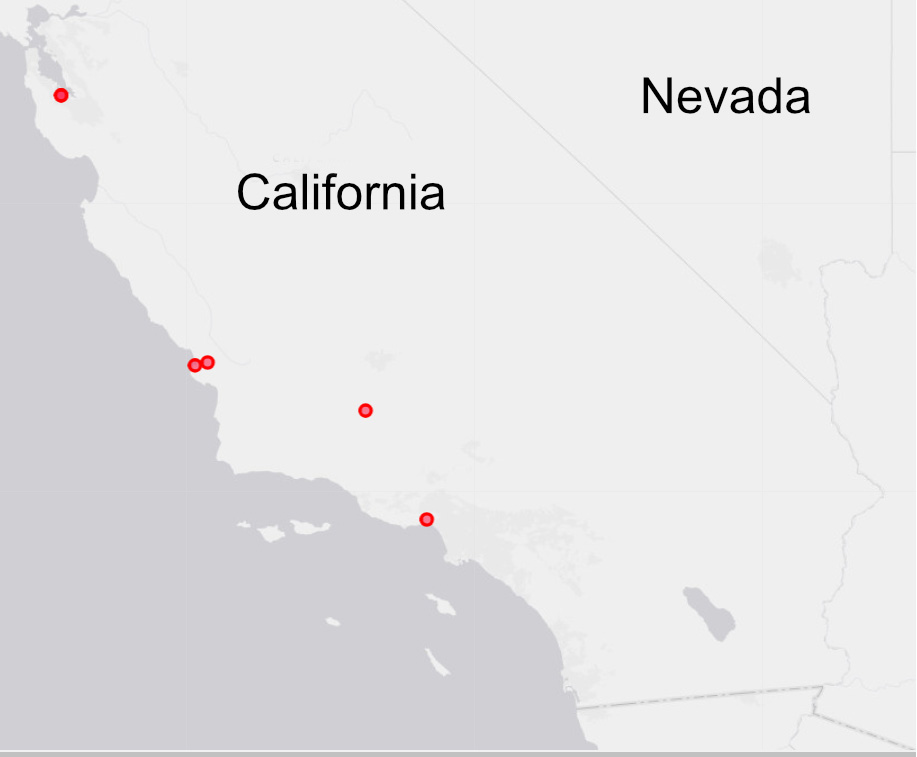
Distribution of *M. simunorum* new species in California.

Known only from California, USA (Fig. 6).

#### Ecology

The habitats at the sites where this species was collected vary from a willow spring in an interior grassland (Wind Wolves), a sycamore/oak forest near a small creek, a coastal floodplain, an old field near the coast, to an inland urban backyard. Many involve at least some exposed water, but this might be simply a reflection of where we put our Malaise traps.

## Supplementary Material

XML Treatment for Megaselia simunorum

## Figures and Tables

**Figure 1.**
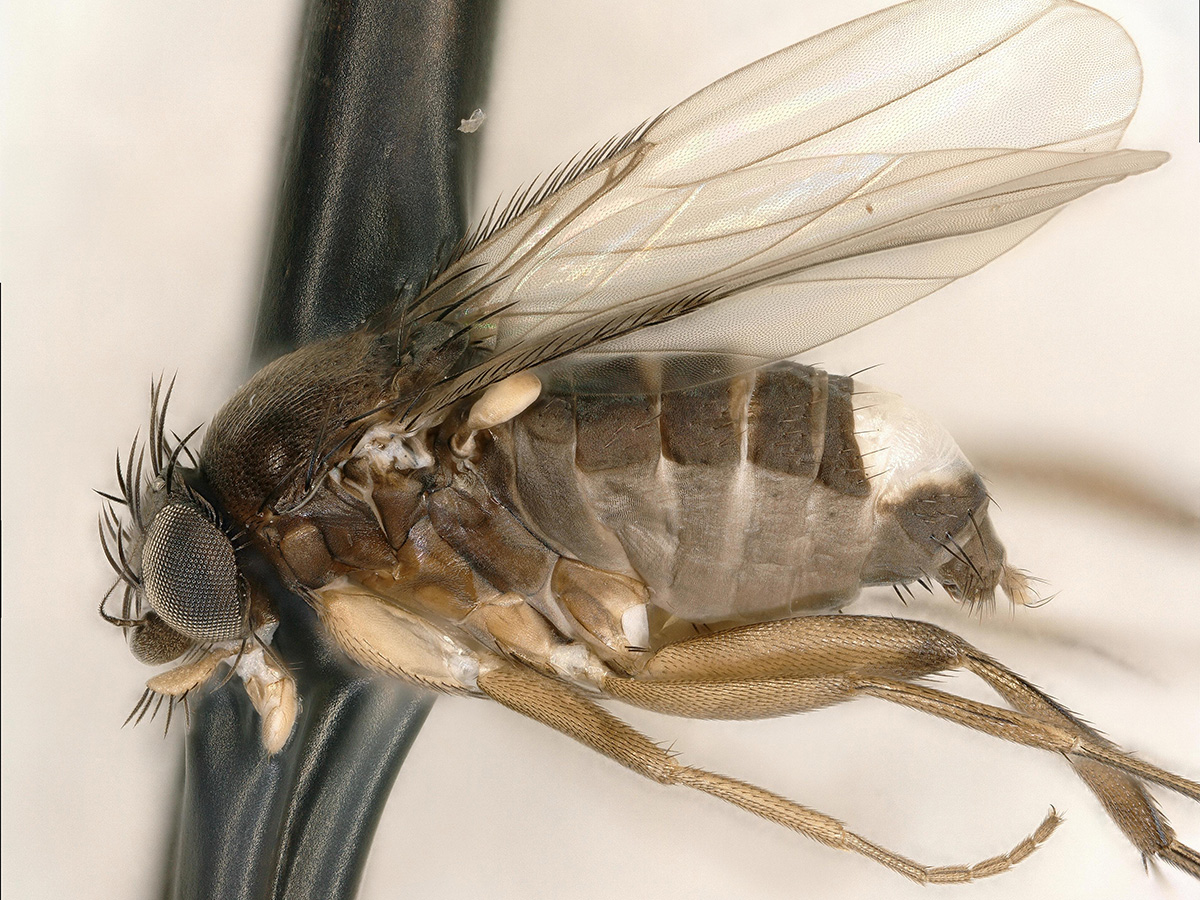
*Megaselia sinumorum* new species, male, lateral.

**Figure 2.**
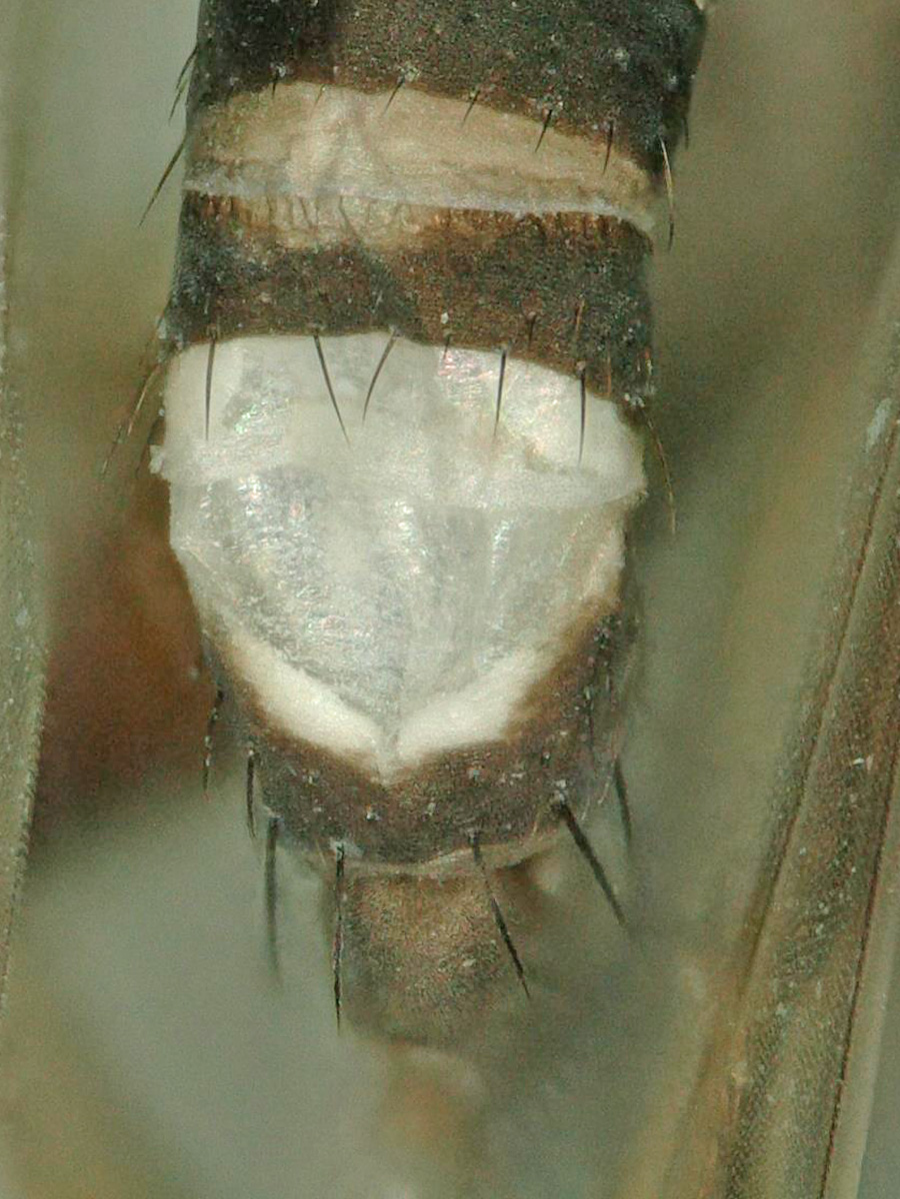
*Megaselia simunorum* new species, male abdomen, dorsal.

**Figure 3.**
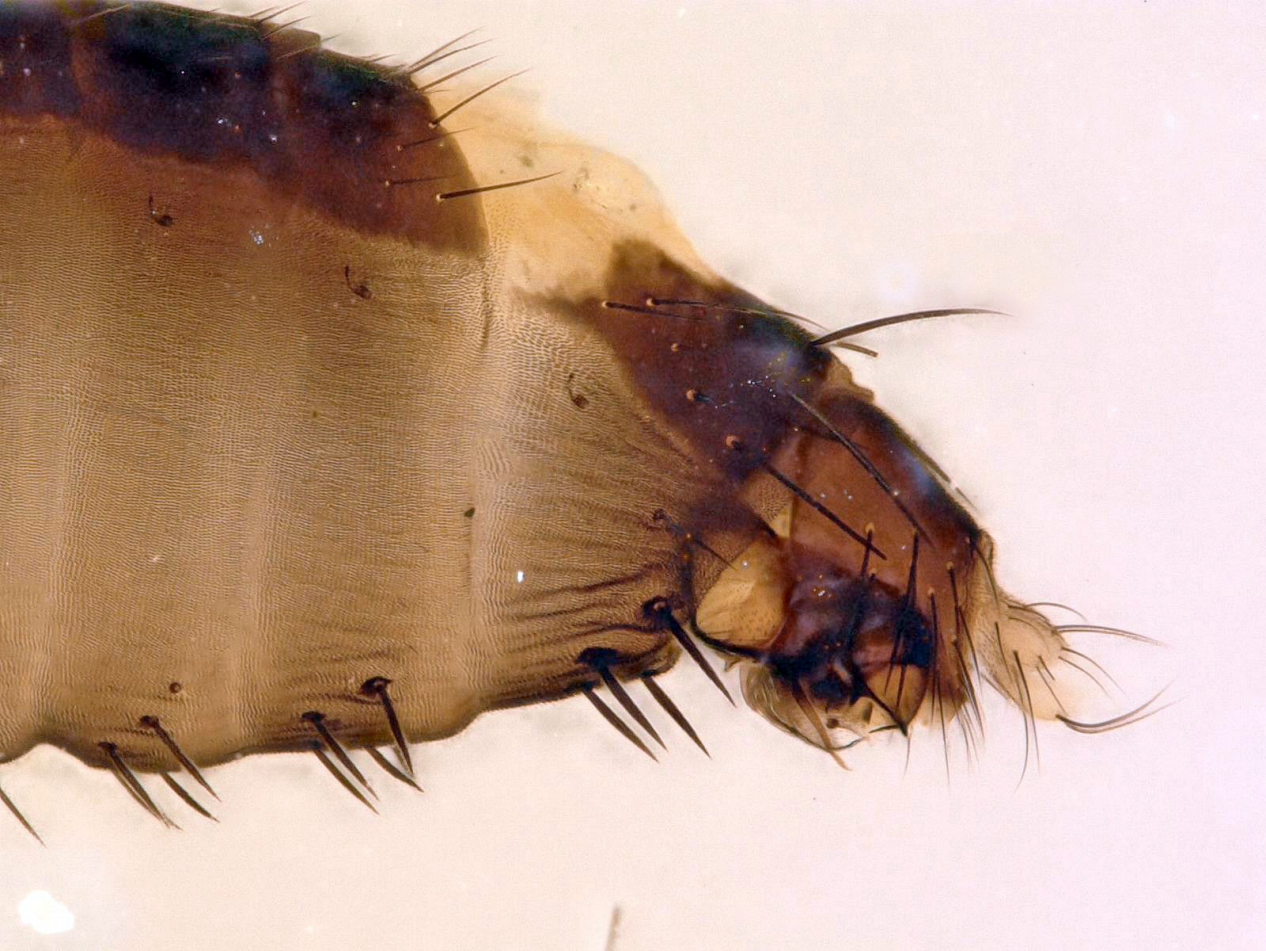
*Megaselia simunorum* new species, male, abdomen lateral.

**Figure 5.**
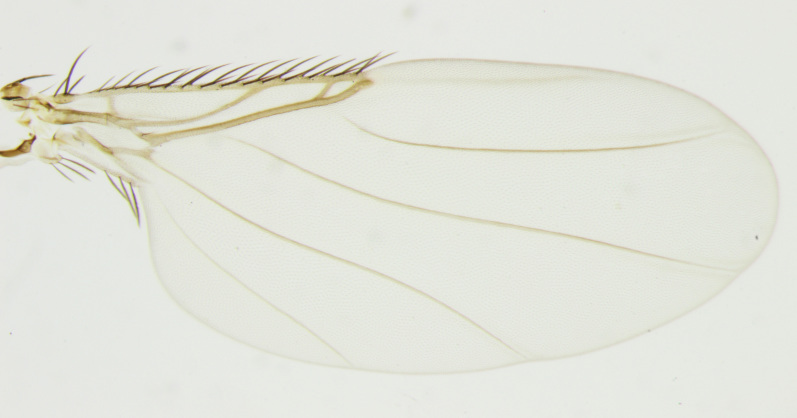
*Megaselia simunorum* new species, wing

**Table 1.** Description of *Megaselia simunorum* new species (Figs 1, 2, 3, 5)

**Head**	
SA ratio	0.42-0.68
VIF position	normal
SPS vesicles	present
Palpal setae length	long
Labellum spinosity	spinose
**Thorax**	
Anepisternum	bare
Relative halter colour	lighter
# NP setae	2
NP cleft	absent
Scutellar setae	2+2
**Leg**	
ts1 palisade	1 to 4
t2 palisade	0.65-0.75
t3 comb bifurcate	absent
t3 setulae	PD only
f3 basal setae	B<AV
f3 basal setae differentiation	absent
**Wing**	Fig. 5
Wing Length (mm)	1.49-1.88
Subcosta	complete (but apically faint)
R seta	long
R2+3	present
Costal index	0.36-0.45
Costal ratios	2.83-3.77: 1.44-2.08: 1
Costal setae length (mm)	0.10-0.14
Number alular setae	3
Alular setae length (mm)	0.09-0.14
Wing colour	lightly infuscated/ clear
**Genitalia**	Fig. 3
AT length	AT<E
E setation	hairs + bristles
General Remarks	Membranous, semi-circular white spot on tergites 5 and 6; abdominal venter with conspicuous plumose setae coming out of sclerotised sockets.
